# The Identification of Multidrug-Resistant Microorganisms including *Bergeyella zoohelcum* Acquired from the Skin/Prosthetic Interface of Amputees and Their Susceptibility to Medihoney™ and Garlic Extract (Allicin)

**DOI:** 10.3390/microorganisms10020299

**Published:** 2022-01-26

**Authors:** Ruby Harsent, Joshua Macleod, Richard S. Rowlands, Paul M. Smith, Neil Rushmere, James Blaxland

**Affiliations:** 1 Llandaff Campus, School of Sport and Health Sciences, Cardiff Metropolitan University, Western Avenue, Cardiff CF5 2YB, UK; ruby.harsent@gmail.com (R.H.); rrowlands@cardiffmet.ac.uk (R.S.R.); nrushmere@cardiffmet.ac.uk (N.R.); 2 Zero2Five Food Industry Centre, Llandaff Campus, Cardiff Metropolitan University, Western Avenue, Cardiff CF5 2YB, UK; st20221179@outlook.cardiffmet.ac.uk; 3 Cyncoed Campus, School of Sport and Health Sciences, Cardiff Metropolitan University, Cyncoed Road, Cardiff CF23 6XD, UK; psmith@cardiffmet.ac.uk

**Keywords:** drug resistance, prosthetic, antimicrobials, medihoney, allicin, *Bergeyella*, Manuka

## Abstract

Users of prosthetic devices face the accumulation of potentially drug-resistant pathogenic bacteria on the skin/prosthesis interface. In this study, we took surface swabs of the skin/prosthesis interface of eleven disabled athletes to identify microorganisms present. In addition to determining their antimicrobial resistance profile, we assessed their sensitivity to Manuka honey and Garlic extract (allicin). Eleven volunteers were directed to swab the skin at the skin/prosthesis interface. After initial isolation of microorganisms, we employed the following general microbiological methods: Gram stain, Catalase test, Oxidase test, lactose fermenting capability, haemolytic capability, Staphaurex, mannitol fermenting capability, Streptex; API Staph, 20E, Candida, and BBL crystal identification system tests. Once identified, isolates were analysed for their sensitivity to penicillin, erythromycin, ampicillin, vancomycin, ceftazidime, ciprofloxacin, gentamicin, and colistin-sulphate. Isolates were also analysed for their sensitivity to allicin (Garlic Extract (GE)) and Manuka honey (Medihoney™) (MH). Eleven isolates were identified*: Bacillus cereus, Staphylococcus haemolyticus, Staphylococcus aureus, Micrococcus luteus, Pseudomonas oryzihabitans, Micrococcus* spp., *Bacillus subtilis, Group D Streptococcus, Pantoea* spp., *Enterobacter cloacae*, and *Bergeyella zoohelcum*. All isolates were resistant to 1 unit of penicillin and 10 μg of ampicillin*. Bergeyella zoohelcum* was observed to have the widest range of resistance with observed resistance against five of the eight antimicrobials employed in this study. This study highlights the prevalence of uncommon drug-resistant microorganisms on the skin within a vulnerable population, highlighting the potential for MH or GE intervention.

## 1. Introduction

It is estimated that there were more than 27,000 amputations in the UK between 2015–2018, with around 176 leg, toe, or foot amputations carried out each week within the UK [1]. The World Health Organisation estimates that the disabled community will continue to rise in proportion with the increase in life expectancy and associated ageing health difficulties [2]. Therefore, there are many amputees that face common problems associated with the use of prostheses, such as malodour and continued infections [3,4]. Amputations are common with the prevalence of chronic wounds caused by vascular diseases such as diabetes mellitus [5], whereby the wounds cannot heal due to inflammation imbalances and infection [6], making recurrent infections especially problematic and leading to a continued risk of further amputation [7]; therefore, the prevention of infections is essential in this group of individuals.

Amputees encounter numerous challenges whilst undertaking physical and sporting activities. This can be due to the increase in heat and moisture that occurs during exercise as a result of ineffective heat transfer inside an enclosed and insulated prosthetic [8]. This not only leads to malodour but creates a suitable environment for microbiological colonisation [3,4,5,6,7,8,9]. One way in which prosthetic users can combat the development of malodour caused by these microorganisms is the use of commercial deodorants or antiperspirant products [10]. Such products traditionally employ antimicrobials, such as triclosan, within their constituents [11]. The use of triclosan and other topical antimicrobials is linked to the development of contact dermatitis and can help develop antimicrobial-resistant microorganisms [12]. Indeed, it has also been shown that certain microorganisms, which are resistant to triclosan, may also have increased resistance to commonly used antibiotics [13]. 

Certain natural products are effective as antimicrobial agents, particularly garlic (allicin) and Manuka honey [14]. Topical applications of Medihoney™ (MH)—a gamma-irradiated Manuka honey occasionally embedded in dressings—has been used extensively in a range of healthcare products [15,16], and for use on chronic wounds such as pressure ulcers and sores [17]. Garlic, specifically one of its bioactive components, allicin (diallyl thiosulfonate), has been used to promote wound healing [18] and has antimicrobial properties both in extracts and volatile components [19]. 

The microflora found on the skin is usually protective [20], and can commonly consist of *Staphylcooccus* spp.; however, when translocated into a wound, infection can occur [21]. Common aetiological agents in a wound infection can consist of endogenous organisms from that area (e.g., from the surface of the skin to the deeper layers of a wound) such as: *Staphylococcus aureus* [22], *Streptococcus pyogenes* [23], and *Candida albicans* [24].

Aetiological agents of wound infections can include both exogenous organisms from the environment and endogenous organisms that have been translocated from another area of the body. Such organisms can consist of Enterococci from the gastrointestinal tract [25], *Clostridium tetani* from the soil and other external environmental sources [26], and *Bacillus cereus,* common in nosocomial surgical infections (especially in the immunocompromised group) [27]. In addition, many fungal infections stem from endogenous fungi, mainly affecting immunocompromised individuals [28]. 

Our objectives were two-fold. Firstly, to isolate and identify some of the potentially pathogenic microorganisms found on the surface of the skin at the skin/prosthesis interface of prosthetic limbs worn by disabled athletes. Secondly, once isolated, we determined the microorganisms’ antimicrobial susceptibility, including their susceptibility to both Manuka honey and allicin.

## 2. Materials and Methods

Control organisms were employed to check the accuracy of the general microbiological methods and consisted of *S. aureus* NCTC 6571, *Lactobacillus acidophilus, Pseudomonas aeruginosa* Pa01*, Escherichia coli* NCTC 10418*, Streptococcus pyogenes* MGAS 6180*, Streptococcus pneumoniae* ATCC 12228*, C. albicans* NCPF 3153 and *S. epidermidis*. Strains were provided by Cardiff Metropolitan University, Cardiff, UK.

Antibiotics: Antibiotic sensitivity discs included: penicillin (1 unit), erythromycin (10 μg), ampicillin (10 μg), vancomycin (30 μg), ceftazidime (10 μg), ciprofloxacin (10 μg), gentamicin (10 μg) and colistin-sulphate (50 μg) and were purchased from Merck, Feltham, UK. 

General laboratory media: All laboratory media and reagents were prepared as per manufacturer’s instructions and were sterilised at 121 °C for 15 min at 15 PSI. 

All tests were performed in triplicate excluding the analytical profile index (API^®^), Biomerieux, USA and BBL™, BD, UK tests. In the case of API and BBL tests, isolates were equilibrated at room temperature (21 ± 1 °C) before tests.

Ethical approval of all procedures associated with participant swabbing was granted by Cardiff Metropolitan University on 20 June 2019 (reference: STA-1115) and for further bacterial identification as part of a postgraduate student project (reference: PGT-2244) on 6 December 2019.

### 2.1. Swab Collection

Using a network of established contacts, eleven participants were recruited with either a single or double lower limb amputation. Participants were asked to swab the skin at the skin/prostheses interface; this was defined as the area of skin directly in contact with the socket and/or liner. A participant information sheet outlined the objectives of the study and detailed the correct method of swabbing and best practice to avoid contamination. Swabs contained a stabilising solution to ensure survival, but not proliferation of isolates, and in all cases, swabs were analysed within 72 h of being obtained. Upon receipt, swabs were assigned random participant number/letter combinations to ensure confidentiality.

### 2.2. Isolate Recovery

Once received, each swab was vortex mixed for 30 s and immediately inoculated onto duplicate tryptone soya agar (TSA) and nutrient agar (NA). Swabs were also inoculated onto Sabouraud agar (SA) (Fisher Scientific, Horsham, UK) plates. Plates were inverted and incubated at 37 °C for 18 h. The morphology of the cultured swab samples and further isolates were assessed according to the Observational Assessment of Cultural Appearance [29] and further isolated into visually pure cultures. Single colonies were sub-cultured onto TSA/SA and incubated as previously described to ensure purity. 

### 2.3. Microbiological Identification Methods 

Following recovery onto solid media agar we employed the following general microbiological methods; Gram stain [30], Catalase test [31], Oxidase test [32], Lactose fermenting capability [33,34], haemolytic capability [33,35], Staphaurex [36,37], mannitol fermenting capability [33,38], Streptex [39,40]; API Staph [41,42], 20E [43,44] and Candida [45,46] and BBL Crystal Identification systems (Gram-positive and Enteric and Non-fermenter) [47,48]. 

### 2.4. Bacterial Standardisation 

A single colony of each isolate was inoculated into 20 mL of sterile tryptone soya broth (TSB) and incubated at 37 °C 18 h. The bacterial suspension was then centrifuged at 5000× *g* for 3 min. The resultant solution was standardised to an optical density of 0.05 at 660 nm; this was calculated to be between 5.8 × 10^6^ and 1.24 × 10^8^ CFU·mL^−1^ for each microorganism tested. This was completed prior to any antimicrobial testing. 

### 2.5. Preparation of Allicin (Garlic Extract) 

Allicin (Fisher Scientific, UK) was prepared by diluting 5000 μg·mL^−1^ (*v*/*v*) Allicin in 9 mL of sterile nutrient broth (NB) to create a 500 μg·mL^−1^ (*v*/*v*) allicin stock solution. This was stored at 3 °C in the dark and used on the day of production. 

### 2.6. Preparation of Medihoney™ Stock

Medical grade Manuka honey (Medihoney™,Comvita^®^, Maidenhead, UK), 2.5 g of honey was homogenised with 2.5 mL of sterile double concentrated NB using a desktop vortex for 2 min at room temperature. Following homogenisation, the stock solution was stored at 4 °C in the dark and used on the day of production. 

### 2.7. Bacterial Inhibition Assays

#### 2.7.1. Determination of Minimum Inhibitory Concentration with Allicin

Allicin was prepared as previously described, along with 190 µL at concentrations of 0, 1.562, 3.125, 6.25, 12.5, 25, 50, 100, 150, 200, 250 and 300 μg·mL^−1^, respectively, before being added to each well within a 96-well plate. In all analyses, a 0 mg mL^−1^ 190 µL growth control (TSB), a media-only (TSB) (sterility check) triplicate well, and a negative growth blank of GE (2nd sterility control) were employed. Analyses were completed in triplicate on three separate occasions. 

#### 2.7.2. Determination of Minimum Inhibitory Concentration with Manuka Honey

Manuka honey was prepared as previously described, along with 190 µL at concentrations of 0, 3, 6.25, 9, 12.5, 15, 25 and 50% *w*/*v*, before being added to each well within a 96 well plate. In all analyses, a 0% (*v*/*v*) 190 µL positive growth control (TSB), a media-only (sterility check) triplicate well and a negative growth blank of MH (2nd sterility control) were employed. Analyses were completed in triplicate on three separate occasions.

### 2.8. Bacterial Inoculation

Isolates were standardised as previously described, and 2 µL of bacterial suspension was inoculated into the 190 µL of the Manuka honey or Garlic suspension in a 96-well plate. The absorbance (660 nm) of each well was measured using a Biotek 800-TS multiplate reader (Agilent, UK) at time 0 and following incubation at 37 °C with shaking at 150 rpm for 24 h. The MIC was calculated by subtracting T = 0 values from the T = 24 h values; a resultant value of less than 0.01 indicated that no growth had occurred.

#### Determination of Minimum Bactericidal Concentration

The bactericidal concentration was determined after 24 h of incubation. From each test well, 50 µL of suspension was aseptically transferred to the surface of a sterile tryptone soya agar plate and spread evenly over the surface using a sterile spreader. Plates were left to dry within a laminar flow cabinet prior to inversion and incubated for 48 h at 37 °C. No growth indicated a bactericidal concentration. 

### 2.9. Antibiotic Sensitivity Testing

Antibiotic sensitivity testing was undertaken by firstly standardising each inoculum as previously described before inoculating 150 µL of each isolate onto the surface of a NA plate and spreading evenly using a sterile spreader. The plates were inverted and incubated for 1 h at 37 °C for 1 h to allow for bacterial adherence and settling; Following incubation, antibiotic discs were added to the surface of the agar as detailed in the Kirby-Bauer Disk Diffusion Susceptibility Test Protocol [49]. Antibiotics employed included penicillin (1 unit), erythromycin (10 μg), ampicillin (10 μg), vancomycin (30 μg), Ceftazidime (10 μg), ciprofloxacin (10 μg), gentamicin (10 μg), and colistin-sulphate (50 μg) and were tested against all isolates to ensure comparisons could be made. Plates were incubated for 24 h in 37 ± 1 °C and resultant zones were measured with a digital calliper (mm). No zone of inhibition indicated resistance (R), and a zone of inhibition > 0 mm indicated sensitivity.

## 3. Results

### 3.1. Participants 

Of the eleven participants that volunteered, nine swabs produced the individual isolates that were taken forward. Participants were all male and aged between 24–60 years of age. The average age of amputation was 23 years of age, whereby five participants reported bilateral amputation above both knees, two participants reported amputation below their right knee, one participant reported amputation below their left knee, and a further participant reported a bilateral congenital defect through both knees. In addition, three participants had reported a previous infection in their amputated limb, yet only two had received treatment with debridement of tissue, antibiotics (flucloxacillin), and in two cases, further bone amputation.

### 3.2. Bacterial Identification

#### 3.2.1. Cultural Appearance

**Figure 1:**
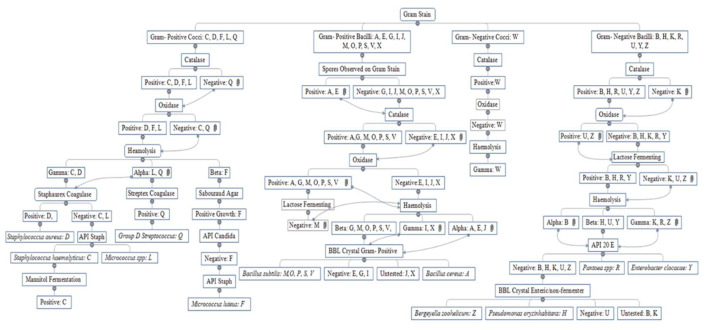
The Identification Process of Nine Isolates. Analysis began with Gram staining, Catalase test, Oxidase test, Lactose fermenting capability, haemolytic capability, Staphaurex latex agglutination test, mannitol fermenting capability, Streptex™ Latex Agglutination Test; with final identification related to API testing kits (Stap, 20E, Candida) and BBL crystal tests. Isolates identified and taken forward for further analysis included; *A: Bacillus cereus, C: Staphylococcus haemolyticus, D: Staphylococcus aureus, F: Micrococcus luteus, H: Pseudomonas oryzihabitans, L: Micrococcus* spp., *P: Bacillus subtilis, Q: Group D Streptococcus, R: Pantoea* spp., *Y: Enterobacter cloacae and Z: Bergeyella zoohelcum*.

Upon observation, the swab samples appeared to contain a range of different colonies, some of which were reoccurring throughout the participant swabs mainly white or yellow convex round mucoid colonies. Results from the Gram stain, catalase, oxidase, lactose fermenting capability, haemolytic capability, Sabouraud agar culture, Staphaurex™, Streptex™, mannitol fermenting capability, API^®^ Staph, API^®^ 20 E, API^®^ Candida, and BBL™ Crystal Identification tests are included in Figure 1. 

Colonies that did not grow successfully or consistently in our selected growth media were removed from subsequent analysis.

#### 3.2.2. MIC and MBC Determination of Manuka Honey 

**Figure 2:**
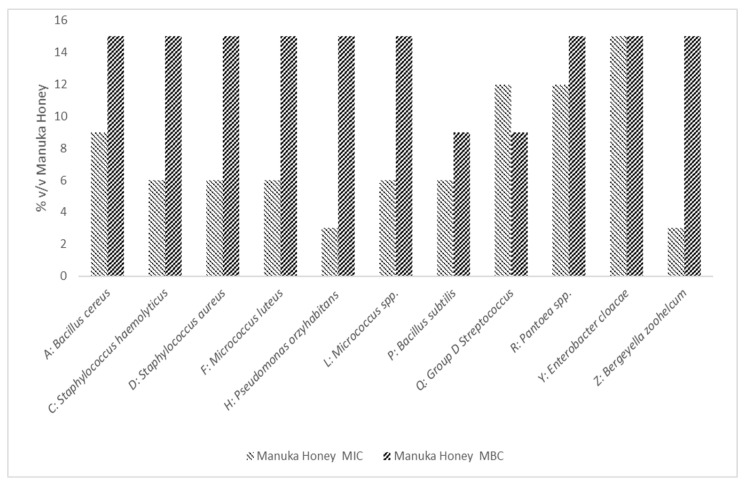
The Minimum Inhibitory and Bactericidal Concentration of Manuka Honey Against *A: Bacillus cereus, C: Staphylococcus haemolyticus, D: Staphylococcus aureus, F: Micrococcus luteus, H: Pseudomonas oryzihabitans, L: Micrococcus* spp., *P: Bacillus subtilis, Q: Group D Streptococcus, R: Pantoea* spp., *Y: Enterobacter cloacae* and *Z: Bergeyella zoohelcum.* Mean MIC 7.63 (*v*/*v*) and Mean MBC 13.90 (*v*/*v*). Results are an average of three separate repeats.

The MIC and MBC of Manuka Honey was Tested Against *A: Bacillus cereus, C: Staphylococcus haemolyticus*, *D: Staphylococcus aureus, F: Micrococcus luteus, H: Pseudomonas oryzihabitans, L: Micrococcus* spp., *P: Bacillus subtilis, Q: Group D Streptococcus, R: Pantoea* spp., *Y: Enterobacter cloacae, and Z: Bergeyella zoohelcum*. We observed that the mean MIC of Manuka honey against our isolates was 7.63 (*v*/*v*) and MBC was 13.90 (*v*/*v*); this is shown in Figure 2. 

#### 3.2.3. MIC and MBC Determination of Allicin

**Figure 3:**
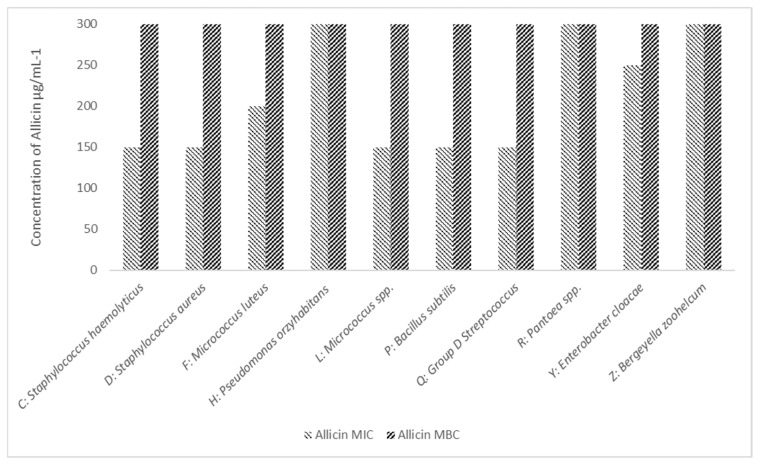
The Minimum Inhibitory and Bactericidal Concentration of Allicin Against *A: Bacillus cereus, C: Staphylococcus haemolyticus, D: Staphylococcus aureus, F: Micrococcus luteus, H: Pseudomonas oryzihabitans, L: Micrococcus* spp., *P: Bacillus subtilis, Q: Group D Streptococcus, R: Pantoea* spp., *Y: Enterobacter cloacae* and *Z: Bergeyella zoohelcum.* We observed inhibition in eight isolates; in one isolate, *B. zoohelcum*, we observed a bactericidal effect, at 200 µg ml^−1^. Results are an average of three separate repeats.

We next investigated the effect of allicin against our isolates. As illustrated in Figure 3, we observed inhibition in eight isolates; *Micrococcus luteus* and *Group D Streptococcus* were not inhibited at the limit of our testing 300 µg·mL^−1^. In only one isolate*, Bergeyella zoohelcum*, did we observe a bactericidal effect, at 200 µg mL^−1^.

### 3.3. Antibiotic Susceptibility 

We investigated the effect of commonly used antibiotics; penicillin (1 unit), erythromycin (10 μg), ampicillin (10 μg), vancomycin (30 μg), ceftazidime (10 μg), ciprofloxacin (10 μg), gentamicin (10 μg) and colistin-sulphate (50 μg). Results are shown in Table 1. We observed that *Pantoea* spp. and *Enterobacter cloacae* were sensitive to the tested antimicrobials apart from; *Pseudomonas oryzihabitans* was resistant to a single antibiotic, whilst all other isolates were resistant to two of the employed antimicrobials. 

## 4. Discussion

Eleven isolates were identified at species level: *B. cereus, B. subtilis, E. cloacae, Micrococcus* spp., *M. luteus, Pantoea* spp., *P. oryzihabitans, S. haemolyticus, S. aureus, Group D Streptococcus,* and *B. zoohelcum*. To the best of our knowledge, *P. oryzihabitans,* and *B. zoohelcum* are not considered to be common colonisers of the skin [27,50]. Interestingly, we did not recover any pathogenic fungi, which may be due to a variety of factors such as a small sample size, medication, or use of a medicated prosthetic material or a good equipment hygiene routine [51,52]. Although some of the isolated organisms could be considered endogenous bacteria, these results suggest that there are a variety of bacteria that are present on the skin and prosthetic devices of prosthesis users, including endogenous skin bacteria, endogenous gastrointestinal bacteria, and exogenous bacteria. 

The isolated species have been shown to cause wound or skin and soft tissue infections; the exogenous and endogenous organisms were all opportunistic nosocomial pathogens that primarily affect immunocompromised patients [27,53,54,55], or patients undergoing surgical procedures, such as an amputation [56,57,58,59], excluding *B. zoohelcum,* which has been isolated from either a cat or dog bite and scratch wounds [60]. Interestingly, this organism has also been observed in patients that have had long term contact with cats and dogs, and have been isolated from patients interacting with therapy dogs [61,62]. 

Worryingly, we found that all isolates were resistant to both penicillin (1 unit) and Ampicillin (10 µg). Only two isolates were sensitive to the cephalosporin, ceftazidime (10 µg). The highest level of resistance was observed in *B. zoohelcum*, which was sensitive only to three antibiotics (vancomycin (30 µg): Ciprofloxacin (10 μg) and Colistin-sulphate (50 μg)) from our panel. This is a similar profile of resistance noted by a previous study by Goldstein and colleagues [63]. All isolates were sensitive to both Colistin-sulphate (50 μg) and Ciprofloxacin (10 μg). 

None of the participants in the study reported an active known infection or had reported the use of any antimicrobials; however, three participants reported a previous infection which had required antibiotic intervention using floxacillin. 

In comparison with the antibiotic treatment, all isolates were sensitive to MH. MH had a bactericidal effect on all isolates with an MBC of 9% *w*/*v* against *Micrococcus luteus* and *Pseudomonas oryzihabitans* and 15% *w*/*v* against all other isolates. In comparison, whilst GE was able to inhibit the growth (25–200 µg) of eight isolates, in only one did we observe a bactericidal effect below 300 µg/mL *B. zoohelcum*.

To the best of our knowledge, there has been no research exploring the use of honey and garlic as antimicrobials against *Pantoea spp.*, *P. oryzihabitans*, *S. haemolyticus*, and *B. zoohelcum*, and this study highlights the potential for the use of either MH or GE against these microorganisms. 

Based on the susceptibility results to the chosen antibiotics, there does not seem to be any explicit link between antibiotic resistance and a higher or lower MIC of MH or GE as concluded in a previous study [64], although this could be further explored using next-generation sequencing. From this data, it is reasonable to suggest that GE and MH do not work by binding to the penicillin-binding proteins in the same way as β-lactams or bind to the aminoacyl-tRNA recognition site, such as aminoglycosides.

This study represents one of the first to explore the microbial diversity of the skin–prosthesis interface and highlights the dangers posed by antimicrobial-resistant microorganisms. Whilst the world is running out of antibiotics, the potential application of natural product-inspired components, such as those derived from honey or plants should be explored in more detail. Results of this study also suggests there is a requirement for comprehensive bacterial identification and increased antimicrobial susceptibility testing on strains not routinely identified in wound infections; here, we isolated the more common microorganisms such as *Staphylococcus*, alongside those which are less common, such as *Bergeyella zoohelcum*. Indeed, *B. zoohelcum* is commonly linked to cat and dog bites, with only five cases of infection reported worldwide [65], though this may be due to the fastidious growth requirements related to variants of the microorganism, leading to systematic underreporting. Despite being multidrug-resistant, the isolate in our study was observed to be the most susceptible in our collection against MH (3/15% *w*/*v* MIC/MBC) and GE (25/200 µg/mL MIC/MBC).

## 5. Conclusions

We conclude that antimicrobial-resistant microorganisms pose a real threat to the general health and wellbeing of individuals who routinely use lower extremity prostheses. Due to the threat of antibiotic resistant microorganisms colonising lower extremity pros-theses, we would encourage the increased investigation into the use of natural product ex-tracts, such as Manuka honey and allicin as potential new and innovative interventions. In light of these findings, it would be pertinent to explore the use of alternative and novel approaches to ensure that the hygiene of an individual’s skin and their prosthetic liners and socket is maintained. Helping reduce the prevalence of multidrug-resistant microorganisms that are present at the skin–prosthesis interface and increasing the general health and wellbeing of those using a prosthetic.

## Figures and Tables

**Table 1:** The Antibiotic Susceptibility Profile of Isolates Against penicillin (1 unit), erythromycin (10 μg), ampicillin (10 μg), vancomycin (30 μg), ceftazidime (10 μg), ciprofloxacin (10 μg), gentamicin (10 μg) and colistin-sulphate (50 μg). All isolates were resistant to 1 unit of penicillin and 10 μg of ampicillin. *Bergeyella zoohelcum* was observed to have the widest range of resistance with observed resistance five of the eight antimicrobials employed in this study. Key: R = Resistance (ZOI = 0 mm); S = Sensitive (ZOI => 0 mm).

	*Isolate ID*										
Susceptibility Profile	*B. cereus*	*S. haemolyticus*	*S. aureus*	*M. luteus*	*Micrococcus* spp.	*B. subtilis*	Group D *Streptococcus*	*P. orzyhabitans*	*Pantoea* spp.	*E. cloacae*	*B. zoohelcum*
Penicillin (1 unit)	R	R	R	R	R	R	R	R	R	R	R
Erythromycin (10 ug)	S	S	S	S	S	S	S	S	R	R	R
Ampicillin (10 ug)	R	R	R	R	R	R	R	R	R	R	R
Vancomycin (30 ug)	S	S	S	S	S	S	S	S	R	R	S
Ceftazidime (10 μg)	R	R	R	R	R	R	R	R	S	S	R
Ciprofloxacin (10 μg)	S	S	S	S	S	S	S	S	S	S	S
Gentamicin (10 μg)	S	S	S	S	S	S	S	S	S	S	R
Colistin-sulphate (50 μg)	S	S	S	S	S	S	S	S	S	S	S

## Data Availability

All data is available from the corresponding author following request.
